# Feasibility of a Generic FFC Intervention for Long-Term Care: Evidence From Interventions in Various Care Settings

**DOI:** 10.1093/geroni/igab046.1616

**Published:** 2021-12-17

**Authors:** Stan Vluggen, Silke Metzelthin, Janneke de Man-van devan Ginkel, Getty Huisman- de Waal, Sandra Zwakhalen

**Affiliations:** 1 Maastricht University, Maastricht, Limburg, Netherlands; 2 UMC Utrecht, Utrecht, Utrecht, Netherlands; 3 Radboud University Medical Center, Nijmegen, Gelderland, Netherlands

## Abstract

Function Focused Care (FFC) interventions support nurses to adapt their level of care to the capabilities of older people and to optimize their self-reliance. Recently, three FFC-interventions were implemented in various Dutch care settings. Lessons learned and implications were synthesized and an advanced FFC-program ‘SELF’ was developed for wide application. SELF comprises interactive and multidisciplinary sessions, is theoretically grounded, primarily focuses on behavior change in nurses, and is tailored to the team’s needs. It also includes policy and environment review, goal-setting, and coaching-on-the-job. SELF was tested in one Dutch psychogeriatric ward. Afterwards, focus groups were conducted with nurses, trainers, manager and coaches. The interactive content, mutual discussions, and practice-based working methods were highly valued. SELF increased awareness and willingness to practice FFC and was considered feasible in practice. Increased involvement and support of allied health professionals and the manager was preferred. A nationwide effectiveness trial is planned after refining SELF.

